# Transactions of the Medical and Chirurgical Faculty of Maryland

**Published:** 1875-10

**Authors:** 


*Transactions of the Medical and Chirurgical Faculty of Maryland,* Seventy-seventh Annual Session, held at Baltimore, April, 1875, Turnbull Brothers, Publishers, Baltimore, Md.

This volume contains the annual oration, "Constitution to the Medical History and Physical Geography of Maryland. By Joseph M. Toner, M. D., of Washington, D. C., and Reports by Drs. T. R. Brown, on Surgery; W. T. Howard, on Obstetrics and Gynæcology; Richard McSherry, on Materia Medica and Therapeutics; W. C. Kloman, on Anatomy and Physiology; G. H. Boyland, on Surgical cases in Foreign Hospitals; J. E. Atkinson, on Contagious Fevers; Saml. Theobald, on Tinnitus Aurium; W. Gleitsmann, on Pulmonary Phthisis; F. T. Miles, on Galvanic Current in Electro Therapeutics; S. C. Chew, on Digitalis in Cardiac Disease; J. Van Bibber, on Treatment of Paralyzed Muscles by Elastic Relaxation; and business of the Society.

---

